# A stiffness-gated YAP-β-catenin axis orchestrates AXIN2 expression in metastatic breast cancer

**DOI:** 10.1016/j.isci.2025.114405

**Published:** 2025-12-11

**Authors:** Yuning Wu, Zhi Su, Chang Ge, Shumaim Barooj, Jeremy A. Hirota, Fei Geng

**Affiliations:** 1 School of Biomedical Engineering, McMaster University, Hamilton, ON, Canada; 2 Firestone Institute for Respiratory Health, Research Institute of St. Joseph’s, Hamilton, ON, Canada; 3 Division of Respirology, Department of Medicine, McMaster University, Hamilton, ON, Canada; 4 Department of Biology, University of Waterloo, Waterloo, ON, Canada; 5 Division of Respiratory Medicine, Department of Medicine, University of British Columbia, Vancouver, BC, Canada

**Keywords:** Mechanobiology, Cell biology, Cancer

## Abstract

Breast cancer progression is strongly influenced by the mechanical properties of the tumor microenvironment, yet how extracellular matrix stiffness coordinates signaling between YAP and β-catenin remains unclear. Using breast cancer cells cultured on soft and stiff 2D substrates and 3D Matrigel spheroids, we show that the rigid matrices drive joint nuclear localization of YAP and β-catenin, whereas compliant environments reveal a compensatory increase in β-catenin nuclear entry following YAP depletion. However, this increase is insufficient to activate canonical Wnt targets, and AXIN2 emerges as a stiffness-sensitive gene requiring cooperative input from both regulators. Cytoskeletal tension and cell density further tune this interplay, indicating that mechanical and architectural cues jointly govern nuclear signaling. In 3D culture, YAP loss reduces β-catenin nuclear localization and spheroid viability in a stiffness-dependent manner. These findings identify a mechanically gated YAP-β-catenin axis that integrates multiple microenvironmental cues to shape transcriptional programs in metastatic breast cancer.

## Highlights

• Stiff substrates drive coordinated nuclear translocation of YAP and β-catenin
• YAP loss enhances β-catenin nuclear entry specifically on soft ECM
• Cytoskeletal tension and cell density gate YAP-β-catenin mechanotransduction
• AXIN2 expression requires YAP under soft conditions but becomes YAP-independent on stiff ECM

## Graphical abstract

**Figure:**
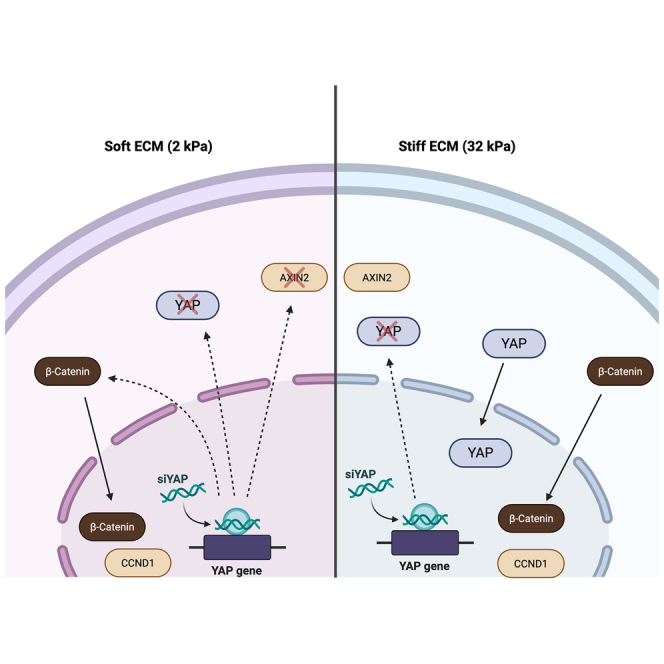

## Introduction

Breast cancer remains one of the leading causes of cancer-related mortality among women worldwide.^1^^,^^2^ The progression of breast cancer from localized tumors to invasive and metastatic disease poses a significant challenge in its management and treatment.^3^^,^^4^^,^^5^ Metastasis, the process by which cancer cells disseminate from the primary tumor site and establish secondary tumors in distant organs, is a complex and multistep phenomenon crucial for breast cancer lethality.^1^^,^^6^^,^^7^

One of the key features that contributes to cancer metastasis is the ability of cancer cells to sense and respond to their microenvironment, which includes the surrounding extracellular matrix (ECM) and neighboring cells.^8^^,^^9^ This process, known as mechanotransduction, involves the conversion of mechanical cues from the ECM into biochemical signals that influence cellular behaviors such as proliferation, migration, and invasion.^10^^,^^11^^,^^12^^,^^13^ Dysregulation of mechanotransduction pathways disrupts tissue homeostasis and promotes cancer progression.^14^

As one of the mechanotransduction pathways, the Hippo pathway is regulated not only by biochemical cues but also by mechanical ones generated from altered cell shape, cell polarity, cell-cell junctions, or cell-ECM adhesion.^14^^,^^15^^,^^16^ Hippo pathway consists of a cascade of kinases that finally controls the phosphorylation of Yes-associated protein (YAP) and its cellular localization.^9^^,^^17^ YAP is phosphorylated and inhibited by the Lats tumor suppressor, and this phosphorylation results in its association with 14–3–3 and cytoplasmic localization of the phosphorylated form of YAP (pYAP).^9^^,^^17^ Unphosphorylated YAP enters the nucleus and mainly interacts with transcription factor TEAD to activate the expression of *CTGF*, promoting cell survival, proliferation, and migration.^9^^,^^17^^,^^18^ YAP has been shown to be the key component in the Hippo pathway and plays crucial roles in the mechanotransduction of metastatic breast cancer,^19^ its interaction with other regulators, such as the Wnt pathway, is critical for the cancer phenotype.^20^ Our recent studies have shown that YAP responds to the changes in ECM stiffness and other mechanical cues of metastatic breast cancer.^21^^,^^22^

As another mechanotransduction pathway, the Wnt/β-catenin pathway is modulated by mechanical stiffness in several cell types, through the differential expression of Wnt ligands, receptors, and by modulating β-catenin levels.^23^^,^^24^ Activation of the Wnt/β-catenin pathway promotes the nuclear translocation of β-catenin, leading to the transcriptional activation of target genes, including *CCND1* and *AXIN2,* involved in cell proliferation and migration.^24^^,^^25^ Aberrant activation of β-catenin has been observed in metastatic breast cancer and is associated with increased tumor aggressiveness and metastatic potential.^26^^,^^27^

Although YAP and β-catenin both contribute to the cellular mechanotransduction, their interaction and synergistic effects in breast cancer metastasis remain unclear. It has been shown that YAP is a component of the β-catenin destruction complex, and YAP is sequestered in the cytoplasm to interact with the destruction complex.^28^^,^^29^^,^^30^ To examine the interaction between β-catenin and YAP in the cellular context of mechanotransduction, we subjected breast cancer cell lines to soft and stiff substrates. Through the manipulation of YAP signaling, cell confluency, and the integrity of the cytoskeleton inside the cells, we revealed a significant compensatory effect of β-catenin to YAP depletion in metastatic breast cancer cells on soft substrate in myosin II dependent, highlighting the critical role of tumor microenvironment (e.g., ECM stiffness) and cytoskeletal organization in regulating cancer phenotype and behaviors.

## Results

### Characterization of Yes-associated protein/β-catenin expression and proliferation rate in breast cancer cells when subject to different stiffness

**Figure 1:**
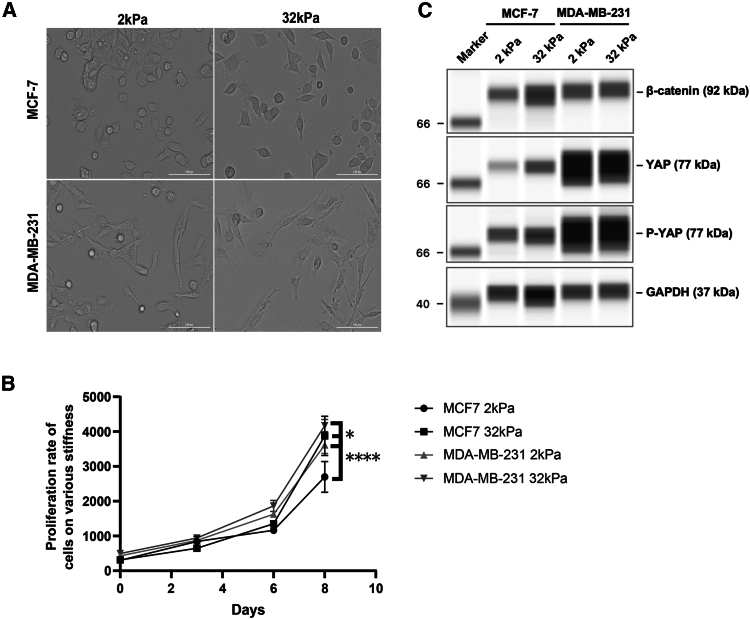
Characterization of YAP/β-catenin expression and proliferation rate in breast cancer cells when subject to different stiffness (A) Random field of view images were taken under brightfield with a magnification of 20× of MCF-7 and MDA-MB-231 cells. Scale bars = 100 μm (B) Proliferation rates of MCF-7 and MDA-MB-231 cells on the substrate with different stiffness were measured using alamarBlue Cell viability reagent over the course of 8 days. Data represent mean ± SD. *n* = 3 wells per condition. Statistical significance was assessed using one-way ANOVA followed by the Tukey post hoc test. ∗*p* < 0.05, ∗∗∗∗*p* < 0.0001. (C) Western blot results of YAP and β-catenin in MCF-7 and MDA-MB-231 cells that were subject to 2 kPa and 32 kPa substrate. Protein expression of β-catenin, YAP, and pYAP was analyzed with GAPDH used for normalization.

In order to investigate the mechanism underlying YAP-β-catenin interplay in breast cancer mechanotransduction in response to substrate stiffness, we cultured MCF-7 and MDA-MB-231 breast cancer cells on CytoSoft Rigidity Plates with elastic moduli of 2 kPa and 32 kPa for over 48 h. As shown in Figure 1A, subtle differences in cell morphology were observed between stiffness conditions (2 kPa and 32 kPa) based on phase-contrast images; however, these observations were qualitative and not quantitatively measured in this study.

Meanwhile, we sought to determine the effects of stiffness on the proliferation rate of both the breast cancer cell lines on the substrate with different elastic moduli. MCF-7 and MDA-MB-231 exhibited higher proliferation rates (Figure 1B) when seeded on the stiff substrate (32 kPa). To better understand the cellular signaling inside those cells associated with the changes in morphology and proliferation, we characterized the expression of three mechanotransduction components, YAP, p-YAP, and β-catenin in MCF-7 and MDA-MB-231 cells that were subject to 2 kPa and 32 kPa substrate (Figure 1C). YAP and phosphorylated YAP (pYAP) expression levels were shown to be much higher in metastatic breast cancer MDA-MB-231 cells than in MCF-7 cells (Figure 1C), which have less metastatic potential.^31^

### Yes-associated protein controls actin cytoskeletal organization and polymerization in a stiffness-dependent manner in metastatic breast cancer cells

**Figure 2:**
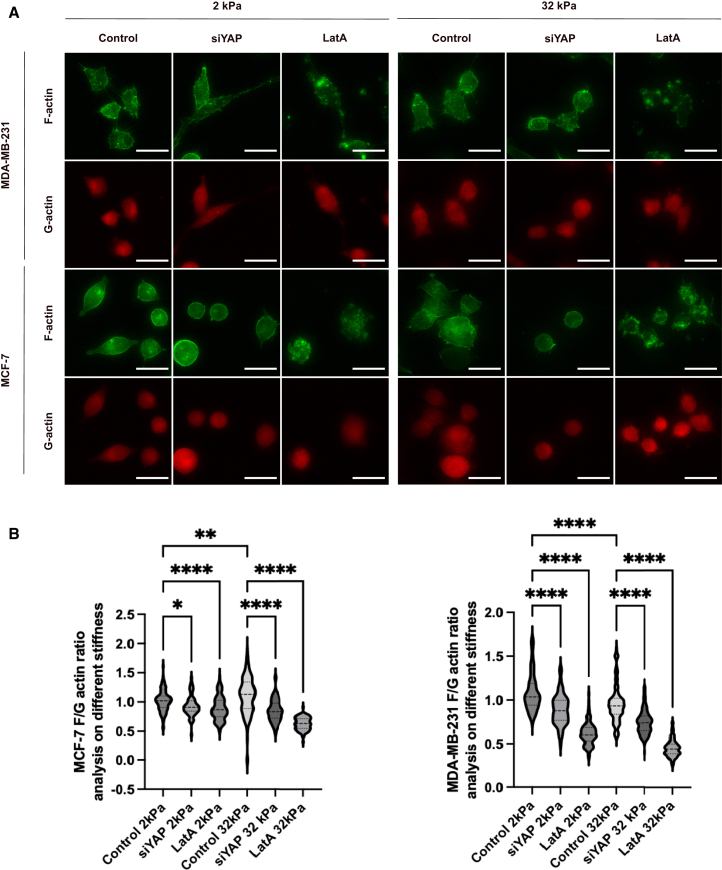
YAP controls actin cytoskeletal organization and polymerization in a stiffness-dependent manner in metastatic breast cancer cells (A) MCF-7 and MDA-MB-231 cells were transfected with non-targeting control siRNAs (control) or siRNA against YAP (siYAP) for 48 h. Control group was treated with LatA at a concentration of 0.4 μM for 1 h (LatA). After the fixation, permeabilization, and blocking, the cells were stained for F-actin and G-actin. Images were taken using Cytation5 under 20× magnification. Scale bars = 50 μm (B) The quantification of the F/G actin ratio in MCF-7 cells (left) and MDA-MB-231 cells (right) from the images in Figure 2A. Image analysis was performed using BioTek Gen5 Software, and bar graphs were created using GraphPad Prism. Data represent mean ± SD. n = individual cells pooled from 5 randomly acquired images per condition. Statistical significance was assessed using one-way ANOVA followed by the Tukey post hoc test. ns: not significant, ∗*p* < 0.05, ∗∗*p* < 0.01, ∗∗∗*p* < 0.001, ∗∗∗∗*p* < 0.0001.

In order to better understand the role of YAP in modulating the mechanotransduction complex that mediates the cellular response to substrate stiffness, we measured the ratio between monomeric globular actin (G-actin) and filamentous actin (F-actin) in cells with or without YAP knockdown (Figure 2). YAP knockdown efficiency in MCF-7 cells was validated by Western blot (Figure S1). To aid in interpreting the changes in actin organization, we included Latrunculin A (LatA), an F-actin depolymerizer that causes F-actin depolymerization by the sequestration of monomeric G-actin.^32^ In comparison, the downregulation of YAP hinders the polymerization of actin in MCF-7 and MDA-MB-231 cells cultured on substrates with both 2 kPa and 32 kPa stiffness, underscoring the involvement of YAP in the formation of actin fibers across different substrate stiffness (Figure 2B). As YAP expression is elevated in MDA-MB-231 cells in comparison to MCF-7 cells (Figure 1), the findings imply a crucial involvement of YAP in regulating the organization of the actin cytoskeleton in metastatic breast cancer cells. Meanwhile, we observed distinct stiffness-dependent cytoskeletal organization between MCF7 and MDA-MB-231 cells. On the stiffer 32 kPa substrate, MCF7 cells exhibited a significantly higher F-/G-actin ratio compared to 2 kPa (Figure 2B), indicating increased actin polymerization and filament formation under elevated mechanical tension. In contrast, MDA-MB-231 cells showed a decreased F-/G-actin ratio on 32 kPa relative to 2 kPa (Figure 2B), suggesting reduced filamentous actin content or increased actin turnover under stiff conditions. Importantly, while the distributions overlap in the violin plots, the mean values are clearly separated, and statistical testing confirmed significance (Figure 2B).

### Enhanced translocation of Yes-associated protein and β-catenin to the nucleus occurs in response to a more rigid substrate in metastatic breast cancer cells

**Figure 3:**
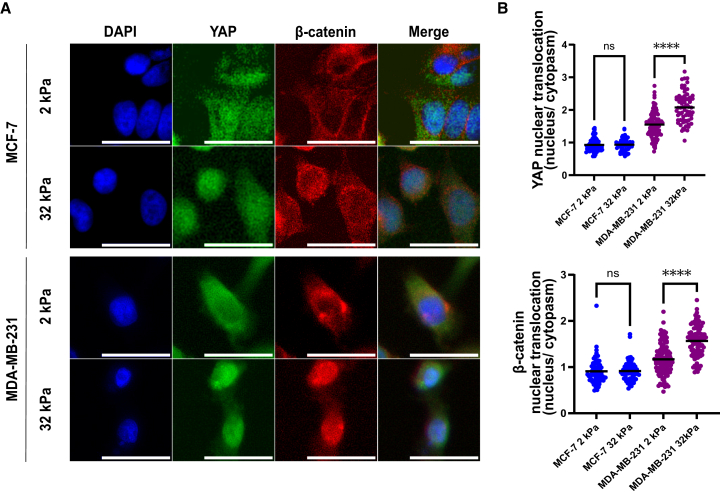
Enhanced translocation of YAP and β-catenin to the nucleus occurs in response to a more rigid substrate in metastatic breast cancer cells (A) The intracellular distribution of β-catenin and YAP in MCF-7 and MDA-MB-231 cells, subjected to the substrates of 2 kPa and 32 kPa, was examined through immunofluorescent staining. Scale bars = 100 μm (B) YAP (top) and β-catenin (bottom) nuclear translocation was measured using BioTek Gen5 Software, and results were generated using GraphPad Prism. Data represent mean ± SD. n = individual cells pooled from 15 images (5 fields × 3 wells) per condition. Statistical significance was assessed using one-way ANOVA followed by the Tukey post hoc test. ns: not significant, ∗∗∗∗*p* < 0.0001.

To delineate the mechanotransduction activity of YAP and β-catenin in response to substrate stiffness, we examined their subcellular localization in MCF-7 and MDA-MB-231 cells cultured on substrates with elastic moduli of 2 kPa and 32 kPa. As shown in Figure 3A, immunofluorescence images revealed that MDA-MB-231 cells exhibited a pronounced increase in the nuclear accumulation of both YAP and β-catenin when cultured on stiff (32 kPa) substrates, compared to soft (2 kPa) substrates. In contrast, MCF-7 cells showed minimal differences in YAP and β-catenin distribution between soft and stiff conditions, with both proteins exhibiting primarily nuclear localization regardless of substrate stiffness. Quantitative analysis in Figure 3B confirmed these observations: the nuclear-to-cytoplasmic ratio of YAP and β-catenin significantly increased in MDA-MB-231 cells in response to 32 kPa stiffness (*p* < 0.0001), while this ratio remained close to 1 in MCF-7 cells under both conditions, indicating no stiffness-dependent translocation. These results underscore the stiffness-sensitive nuclear translocation of YAP and β-catenin as a hallmark of metastatic breast cancer cells, linking substrate rigidity to the activation of a coordinated mechanotransduction network.

### Yes-associated protein knockdown enhances β-catenin nuclear translocation on soft, but not stiff, substrates in metastatic breast cancer cells

**Figure 4:**
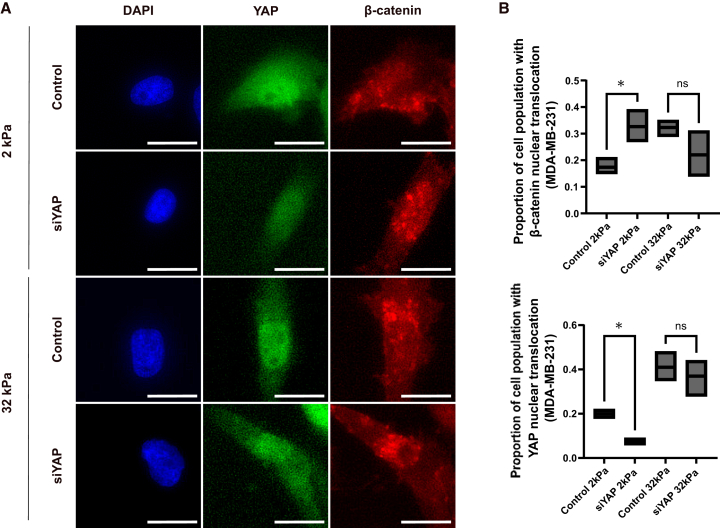
YAP knockdown enhances β-catenin nuclear translocation on soft, but not stiff, substrates in metastatic breast cancer cells (A) The analysis of YAP and β-catenin expression in MDA-MB-231 cells upon YAP knockdown via immunofluorescent staining. Scale bars = 100 μm (B) The quantitative determination of nuclear translocation of β-catenin (top graph) and YAP (bottom graph) in MDA-MB-231 cells that were subjected 2 kPa and 32 kPa following the transfection of non-targeting siRNA (control) or siRNA against YAP (siYAP). *n* = 3 wells per condition. Data represent mean ± SD. Statistical significance was assessed using one-way ANOVA followed by the Tukey post hoc test. ns: not significant, ∗*p* < 0.05.

Given the effect of substrate stiffness on the nuclear localization of YAP and β-catenin, we investigated their interplay by conducting YAP knockdown using siRNA in MDA-MB-231 cells, which are more responsive to stiffness cues compared to MCF-7 cells. As shown in Figure 4A, immunofluorescence staining revealed that YAP knockdown effectively reduced YAP expression in both soft (2 kPa) and stiff (32 kPa) substrates. Notably, under soft substrate conditions (2 kPa), siYAP-treated cells exhibited a striking increase in the nuclear accumulation of β-catenin compared to the control group, as visualized by intense red nuclear fluorescence in β-catenin channels. In contrast, under stiff conditions (32 kPa), β-catenin localization remained predominantly cytoplasmic regardless of YAP knockdown, indicating a lack of nuclear translocation.

Quantitative analysis in Figure 4B confirmed these observations: β-catenin’s nuclear/cytoplasmic ratio significantly increased upon YAP depletion under soft conditions (*p* < 0.05), while no statistical difference was observed on stiff substrates. Meanwhile, the nuclear/cytoplasmic ratio of YAP itself was markedly reduced in siYAP-treated cells on a 2 kPa substrate instead of a 32 kPa substrate (Figure 4B). These findings highlight a stiffness-dependent compensatory mechanism, wherein YAP depletion promotes β-catenin nuclear translocation specifically under soft substrate. This interplay suggests that ECM stiffness acts as a critical modulator of YAP-β-catenin crosstalk in metastatic breast cancer cells.

### β-catenin nuclear translocation following Yes-associated protein knockdown is regulated by cell density in metastatic breast cancer cells

**Figure 5:**
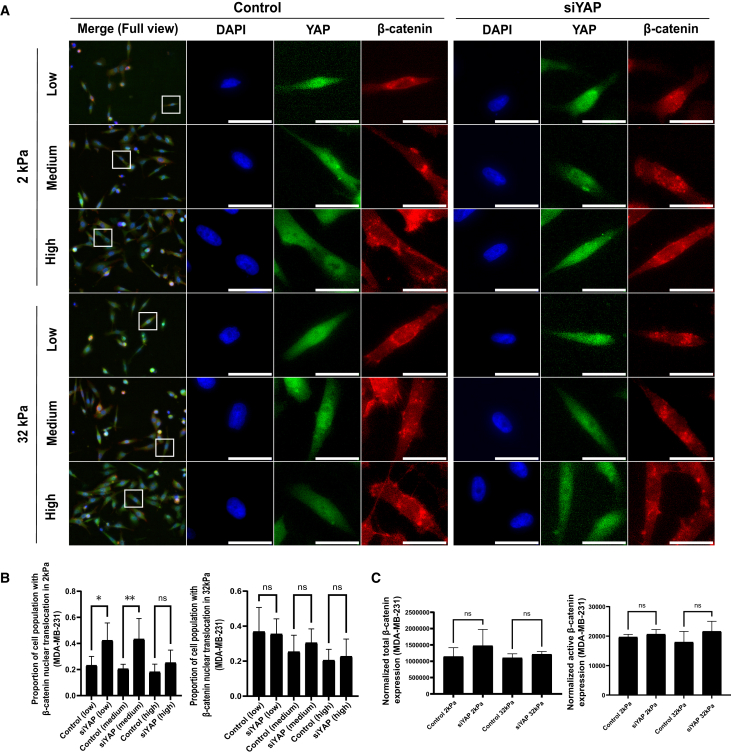
β-catenin nuclear translocation following YAP knockdown is regulated by cell density in metastatic breast cancer cells (A) YAP and β-catenin in MDA-MB-231 cells at low, medium, and high confluency after YAP knockdown. Low confluency (30% confluency), medium confluency (50% confluency), and high confluency (80% confluency). MDA-MB-231 cells that were subject to 2 kPa and 32 kPa substrates were transfected with non-targeting siRNA (control) or siRNA against YAP (siYAP) before the immunofluorescent staining for YAP and β-catenin. Scale bars = 100 μm (B) The percentages of the cell population that show β-catenin nuclear translocation in Figure 5A were measured in Agilent BioTek Gen 5 and plotted in GraphPad Prism. Data represent mean ± SD. *n* = 3 wells per condition. Statistical significance was assessed using one-way ANOVA followed by the Tukey post hoc test. ns: not significant, ∗*p* < 0.05, ∗∗*p* < 0.01. (C) The analysis of β-catenin and its active isoform expression in MCF-7 and MDA-MB-231 cells on 2 kPa and 32 kPa substrates following YAP knockdown. Total protein amount was used for normalization, and bar graphs were generated using GraphPad Prism. Data represent mean ± SD. *n* = 3 wells per condition. Statistical significance was assessed using one-way ANOVA followed by the Tukey post hoc test. ns: not significant.

To further dissect the regulatory dynamics between YAP and β-catenin, we investigated how cell confluency influences β-catenin nuclear translocation in response to YAP knockdown in MDA-MB-231 cells. Cells were seeded at defined densities to establish low (∼30%), medium (∼50%), and high (∼80%) confluency prior to siRNA-mediated YAP depletion. As shown in Figure 5A, immunofluorescence staining revealed that under low and medium confluency on 2 kPa substrates, YAP knockdown markedly increased β-catenin nuclear localization, with a stronger nuclear β-catenin signal observed compared to control. However, at high confluency, β-catenin distribution remained largely cytoplasmic regardless of YAP status. Similarly, on 32 kPa substrates, β-catenin localization was minimally affected by YAP depletion across all confluency levels.

Quantification of these observations in Figure 5B demonstrated that the percentage of β-catenin nuclear-translocated cells nearly doubled following YAP knockdown in low and medium confluency on soft substrate (2 kPa), whereas no significant differences were observed under high confluency or stiff substrate conditions (32 kPa).

To validate these findings at the protein level, we performed capillary-based immunoblotting to assess total and active β-catenin levels under high confluency conditions (Figure 5C). Consistent with the imaging data, Western blot analysis confirmed that YAP knockdown did not lead to significant changes in β-catenin or active β-catenin expression under high confluency, reinforcing the conclusion that cell density modulates β-catenin responsiveness to YAP signaling. Collectively, these results suggest that β-catenin nuclear translocation upon YAP depletion is most pronounced under low-to-intermediate cell densities and compliant ECM conditions, revealing a finely tuned interplay between mechanical and density cues in metastatic breast cancer cells.

### β-catenin nuclear translocation upon Yes-associated protein depletion supports cell proliferation and migration in metastatic breast cancer cells in a stiffness-dependent manner

Given the observed responsiveness of β-catenin nuclear translocation to YAP depletion (Figure 4), we next sought to investigate how this molecular shift impacts cell behavior, specifically proliferation and migration. We employed two functional assays: the alamarBlue proliferation assay and a gap closure wound healing assay, to quantitatively assess cell viability and motility under different stiffness conditions (2 kPa vs. 32 kPa) and siRNA treatments (control, siYAP, and siYAP+siβ-catenin).

**Figure 6:**
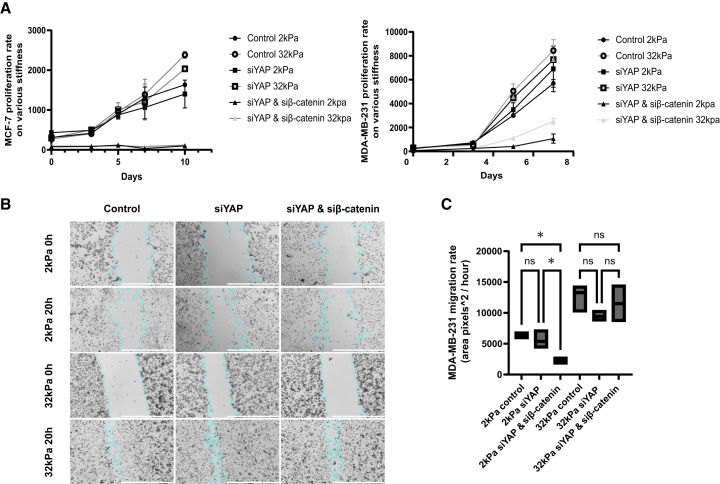
β-catenin nuclear translocation upon YAP depletion supports cell proliferation and migration in metastatic breast cancer cells in a stiffness-dependent manner (A) Proliferation analysis of MCF-7 and MDA-MB-231 cells following YAP knockdown. Both MCF-7 cells and MDA-MB-231 cells were transfected with non-targeting siRNA (control), siRNA against YAP (siYAP), or siRNA against β-catenin (siβ-catenin). Then alamarBlue assay was performed for a period of 7 or 10 days at each time point for both MCF-7 cells and MDA-MB-231 cells. Then the data were generated using GraphPad Prism. Data represent mean ± SD. *n* = 3 wells per condition. (B) Cell migration analysis of MDA-MB-231 cells that were cultured on 2 kPa or 32 kPa substrates before the transfection with non-targeting siRNA (control), or siRNA against YAP (siYAP), or siRNA against YAP and siRNA against β-catenin (siYAP and siβ-catenin). Then the gap closure assay was performed, and the gap areas were imaged using Cytation5 under 4× magnification at 0 h and 20 h. Scale bars = 1000 μm (C) Quantitative analysis of the cell migration assay in Figure 6B was conducted using ImageJ, and the results were generated using GraphPad Prism. Data represent mean ± SD. *n* = 3 wells per condition. Statistical significance was assessed using one-way ANOVA followed by the Tukey post hoc test. ns: not significant, ∗*p* < 0.05.

As shown in Figure 6A, MDA-MB-231 cells displayed increased proliferation on stiff (32 kPa) substrates compared to soft (2 kPa) conditions. YAP knockdown alone had minimal effect on proliferation under both stiffness conditions, suggesting potential compensation by other pathways—most notably β-catenin. However, dual knockdown of YAP and β-catenin led to a significant reduction in proliferation only on soft substrate (2 kPa), while cells on stiff substrates maintained their proliferative capacity.

To assess migratory behavior, we performed a gap closure assay visualized in Figure 6B. Representative images revealed efficient wound healing in control and siYAP-treated cells across both stiffness conditions. However, siYAP+siβ-catenin cells cultured on 2 kPa substrates demonstrated a marked delay in gap closure at 20 h, reflecting impaired migration.

Quantitative analysis of wound closure, presented in Figure 6C, confirmed that MDA-MB-231 cells on 2 kPa substrates exhibited significantly reduced migration rates upon double knockdown compared to single knockdown or control. In contrast, migration on 32 kPa substrates remained unaffected by either YAP or dual YAP/β-catenin depletion, indicating that substrate stiffness buffers against the loss of these signaling components in regulating motility.

These findings support the notion that β-catenin compensates for YAP loss under compliant conditions. This compensatory mechanism appears critical for maintaining both proliferation and migration on soft ECM but becomes redundant on stiff substrates where alternative mechanotransduction pathways likely dominate.

### Myosin II activity facilitates Yes-associated protein and β-catenin nuclear translocation, while intact actin filaments restrict β-catenin nuclear localization

To further elucidate the cytoskeletal mechanisms underlying the YAP-β-catenin regulatory axis, we examined how inhibiting cytoskeletal components influences β-catenin nuclear translocation after YAP knockdown. MDA-MB-231 cells were cultured on soft (2 kPa) substrates and subjected to siRNA-mediated YAP depletion. To perturb cytoskeletal function, cells were treated with Blebbistatin, a specific myosin II inhibitor, or LatA, which disrupts actin filament polymerization.

**Figure 7:**
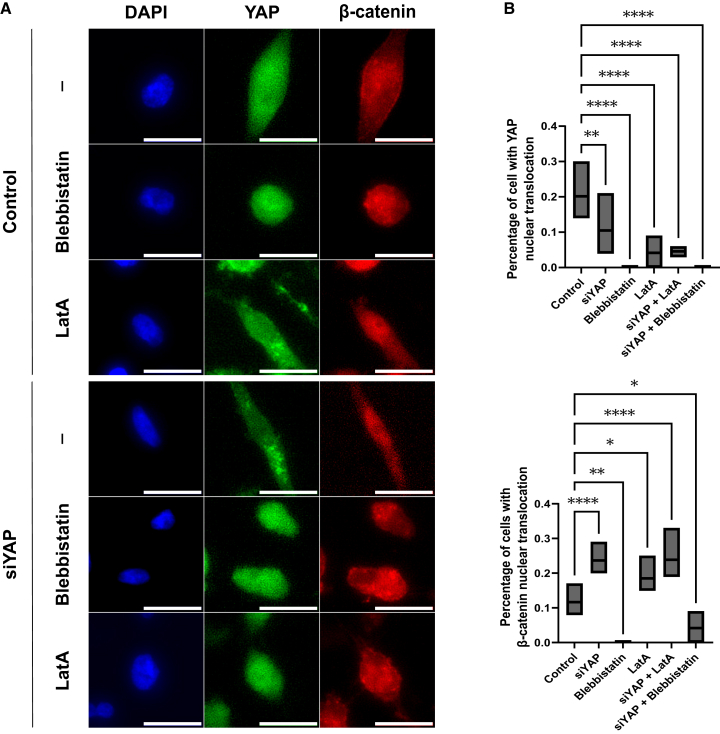
Myosin II activity facilitates YAP and β-catenin nuclear translocation, while intact actin filaments restrict β-catenin nuclear localization (A) MDA-MB-231 cells were transfected with non-targeting siRNA (control), siRNA against YAP (siYAP). Control or siYAP cells were treated with Blebbistatin at a concentration of 50 μM for 30 min or with LatA at a concentration of 0.4 μM for 1 h before the immunofluorescence staining for YAP and β-catenin. The images were taken using Cytation5 under 20× magnification. Scale bars = 100 μm (B) Cell populations with β-catenin nuclear translocation in Figure 7A were measured using BioTek Gen5 Software, and the results were generated using GraphPad Prism. Data represent mean ± SD. *n* = 3 wells per condition. Statistical significance was assessed using one-way ANOVA followed by the Tukey post hoc test. ns: not significant, ∗*p* < 0.05, ∗∗*p* < 0.01, and ∗∗∗∗*p* < 0.0001.

As shown in Figure 7, YAP knockdown under vehicle-treated conditions led to a marked increase in nuclear β-catenin in MDA-MB-231 cells. Inhibition of myosin II with Blebbistatin significantly reduced the nuclear localization of both β-catenin and YAP, indicating that myosin II-mediated contractility is essential for their nuclear translocation. In contrast, the disruption of actin polymerization using LatA alone resulted in elevated nuclear β-catenin levels (Figure 7B). This suggests that actin depolymerization alone is sufficient to drive maximal β-catenin nuclear accumulation, and that the compensatory effect of YAP loss operates through a mechanism that converges with or is overridden by actin filament disruption.

### Substrate stiffness modulates total and phosphorylated β-catenin levels in breast cancer cells

**Figure 8:**
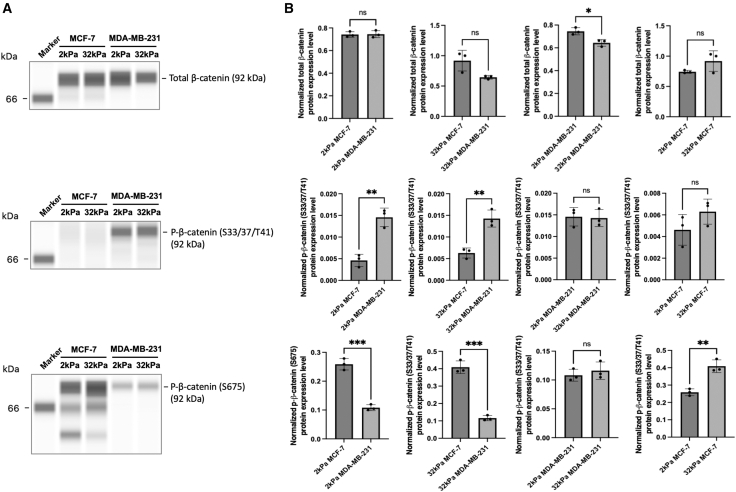
Matrix stiffness modulates total and phosphorylated β-catenin levels in breast cancer cells (A) Representative Western blots of total β-catenin and phosphorylated β-catenin at S33/S37/T41 and S675 in MCF-7 and MDA-MB-231 cells cultured on soft (2 kPa) and stiff (32 kPa) substrates. (B) Quantification of protein levels normalized to loading controls (mean ± SD). Top row: Total β-catenin expression showed no significant differences across stiffness in MCF-7 cells but was significantly increased in MDA-MB-231 cells cultured on stiff (32 kPa) compared to soft (2 kPa) substrates (∗*p* < 0.05). Middle row: Phosphorylation of β-catenin at S33/S37/T41 (associated with degradation) was significantly higher in MDA-MB-231 cells than in MCF-7 cells at both stiffness levels (∗∗*p* < 0.01), indicating enhanced β-catenin degradation in the metastatic subtype. However, substrate stiffness had no significant effect on p-β-catenin (S33/S37/T41) within either cell line. Bottom row: Phosphorylation at S675 (associated with nuclear translocation) was significantly higher in MCF-7 cells than in MDA-MB-231 cells at both stiffness levels ∗∗∗*p* < 0.001). Data represent mean ± SD. *n* = 3 wells per condition. Statistical significance was assessed using two-tailed t-tests. ns: not significant; ∗*p* < 0.05, ∗∗*p* < 0.01, ∗∗∗*p* < 0.001.

To investigate how substrate stiffness modulates the YAP-dependent regulation of downstream Wnt signaling components, we examined β-catenin protein levels and phosphorylation status in MCF-7 and MDA-MB-231 cells cultured on soft (2 kPa) and stiff (32 kPa) substrates. As shown in Figure 8A, total β-catenin expression remained consistent across stiffness conditions in MCF-7 cells. In contrast, MDA-MB-231 cells exhibited a significant decrease in total β-catenin expression on stiff substrates compared to soft ones, suggesting a stiffness-sensitive regulation of β-catenin protein stability in this metastatic subtype. Quantitative analysis in Figure 8B confirmed that the difference in total β-catenin expression between 2 kPa and 32 kPa was statistically significant in MDA-MB-231 cells but not in MCF-7 cells.

We further evaluated the phosphorylation of β-catenin at S33/S37/T41, which promotes degradation, and S675, which is associated with nuclear translocation. Phosphorylation at S33/S37/T41 was consistently higher in MDA-MB-231 cells compared to MCF-7 cells under both stiffness conditions, with significant differences observed at 2 kPa, indicating enhanced β-catenin degradation in the aggressive, mechanosensitive MDA-MB-231 line. However, substrate stiffness did not significantly change S33/S37/T41 phosphorylation within either cell line. In contrast, phosphorylation at S675 was significantly enriched in MCF-7 cells relative to MDA-MB-231 cells at both stiffness levels, consistent with greater potential for nuclear translocation in the luminal subtype. Notably, stiff substrate conditions suppressed S675 phosphorylation in MCF-7 cells (Figure 8B), suggesting that high stiffness may attenuate β-catenin nuclear signaling in these cells.

Figure 8 demonstrates that β-catenin is regulated by both substrate stiffness and cell type. In MDA-MB-231 cells, stiff substrates reduce total β-catenin protein levels and enhance phosphorylation at degradation-associated sites (S33/37/T41).

### 3D spheroid model reveals the stiffness-dependent regulation of Yes-associated protein and β-catenin localization

**Figure 9:**
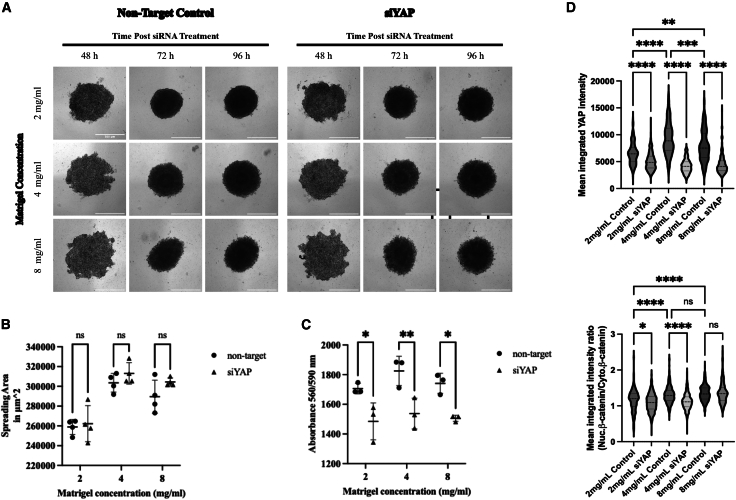
3D spheroid model reveals the stiffness-dependent regulation of YAP and β-catenin localization (A) Bright-field images of MDA-MB-231 spheroids grown in 3D Matrigel at 2, 4, and 8 mg/mL for 48–96 h post-siRNA transfection. Scale bars = 500 μm. (B) Quantification of spheroid spreading area shows no significant changes following YAP knockdown. Data represent mean ± SD. *n* = 4 wells per condition. Statistical significance was assessed using two-way ANOVA followed by the Tukey post hoc test. ns: not significant. (C) Cell viability measured by absorbance indicates a significant reduction in proliferation at all stiffness levels upon YAP knockdown. Data represent mean ± SD. *n* = 3 wells per condition. Statistical significance was assessed using two-way ANOVA followed by the Tukey post hoc test. ∗*p* < 0.05, ∗∗*p* < 0.01. (D) Violin plots show reduced YAP expression and β-catenin nuclear-to-cytoplasmic ratio in siYAP-treated spheroids. The effect on β-catenin localization is stiffness-dependent, observed at 2 and 4 mg/mL but not at 8 mg/mL. Data represent mean ± SD. n = individual cells pooled from 15 images (5 fields × 3 wells) per condition. Statistical significance was assessed using one-way ANOVA followed by the Tukey post hoc test. ns: not significant, ∗*p* < 0.05, ∗∗∗∗*p* < 0.0001.

To further explore the YAP-β-catenin relationship in a physiologically relevant environment, we employed a 3D Matrigel-based spheroid model of MDA-MB-231 cells cultured in matrices of increasing stiffness (2, 4, and 8 mg/mL Matrigel). As shown in Figure 9A, spheroids formed compact aggregates at all concentrations, and YAP knockdown did not visibly alter spheroid morphology across the time points of 48, 72, and 96 h. Quantification of the spheroid spreading area in Figure 9B confirmed these observations, showing no statistically significant differences between control and siYAP groups at any Matrigel concentration, indicating that YAP depletion does not affect physical spheroid expansion. No significant difference in spheroid circularity or aspect ratio was observed between control and siYAP-treated spheroids (Figure S2).

However, cell viability was significantly impaired by YAP knockdown, as revealed by absorbance-based alamarBlue viability assays in Figure 9C. Across all stiffness levels (2, 4, and 8 mg/mL), siYAP-treated spheroids exhibited a marked reduction in metabolic activity compared to controls, suggesting that YAP plays a critical role in maintaining proliferative capacity in 3D culture regardless of ECM stiffness.

To assess molecular responses, we conducted immunofluorescence staining for YAP and β-catenin. As shown in Figure 9D (upper panel), YAP expression was significantly decreased in siYAP-treated spheroids at all Matrigel concentrations, confirming effective knockdown in the 3D environment. This downregulation closely mirrors our 2D results and validates siRNA efficacy in both culture systems. In Figure 9D (lower panel), we examined the nuclear-to-cytoplasmic ratio of β-catenin. Notably, YAP knockdown significantly reduced β-catenin nuclear localization at 2 and 4 mg/mL Matrigel, while the ratio remained unchanged at 8 mg/mL. This indicates that β-catenin nuclear translocation in response to YAP loss is modulated by matrix stiffness and becomes refractory at higher stiffness.

### Yes-associated protein knockdown selectively downregulates AXIN2 expression on soft substrates in metastatic breast cancer cells

**Figure 10:**
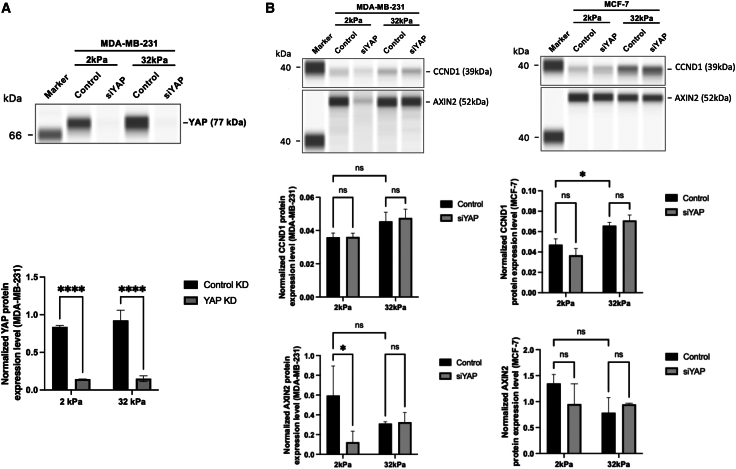
YAP knockdown selectively downregulates AXIN2 expression on soft substrates in metastatic breast cancer cells (A) Capillary-based immunoblot and quantification confirming efficient YAP knockdown in MDA-MB-231 cells cultured on soft (2 kPa) and stiff (32 kPa) substrates for 48 h. YAP protein levels were significantly reduced in siYAP-treated cells compared to control (∗∗∗∗*p* < 0.0001). Data represent mean ± SD. *n* = 3 wells per condition. Statistical significance was assessed using two-way ANOVA followed by the Tukey post hoc test. (B) Immunoblot and quantification of CCND1 and AXIN2 protein expression in MDA-MB-231 and MCF-7 cells following YAP knockdown. In MDA-MB-231 cells, YAP depletion did not significantly affect CCND1 expression at either stiffness, while AXIN2 levels were significantly decreased under 2 kPa (∗*p* < 0.05) but not 32 kPa. In MCF-7 cells, CCND1 expression was significantly elevated on stiff substrate compared to soft (∗*p* < 0.05), independent of YAP knockdown. AXIN2 expression in MCF-7 cells was not significantly affected by YAP knockdown at either stiffness. Data represent mean ± SD. *n* = 3 wells per condition. Statistical significance was assessed using two-way ANOVA followed by the Tukey post hoc test. ns: not significant.

We next assessed how substrate stiffness and cell type influence β-catenin expression and its post-translational modifications by analyzing MDA-MB-231 and MCF-7 cells cultured on soft (2 kPa) and stiff (32 kPa) substrates. As shown in Figure 10A, capillary-based immunoblotting confirmed that YAP knockdown was highly effective in MDA-MB-231 cells, with a dramatic reduction in YAP protein levels at both stiffness conditions (∗∗*p* < 0.0001), thereby validating the experimental setup.

In Figure 10B, we examined the expression of canonical Wnt targets CCND1 and AXIN2. In MDA-MB-231 cells, CCND1 protein expression remained unchanged following YAP knockdown at both 2 kPa and 32 kPa, indicating that this gene is not responsive to YAP in this context. However, AXIN2 protein levels were significantly reduced upon YAP knockdown on the soft (2 kPa) substrate (∗*p* < 0.05), while no change was observed on the stiff (32 kPa) substrate. This suggests that AXIN2 is a stiffness-sensitive downstream target of YAP specifically in the metastatic MDA-MB-231 line.

In contrast, MCF-7 cells exhibited different regulation. As shown in Figure 10B (right panels), YAP knockdown did not significantly alter AXIN2 expression at either stiffness level, further reinforcing the idea that AXIN2 regulation by YAP is cell type-specific. Interestingly, CCND1 protein levels in MCF-7 cells were significantly elevated under stiff conditions compared to soft, but this difference was independent of YAP knockdown (∗*p* < 0.05). This indicates that stiffness alone influences CCND1 expression in luminal breast cancer cells, potentially via YAP-independent mechanisms.

### β-catenin knockdown reduces CTGF mRNA but not protein expression, and does not affect CCND1 expression

**Figure 11:**
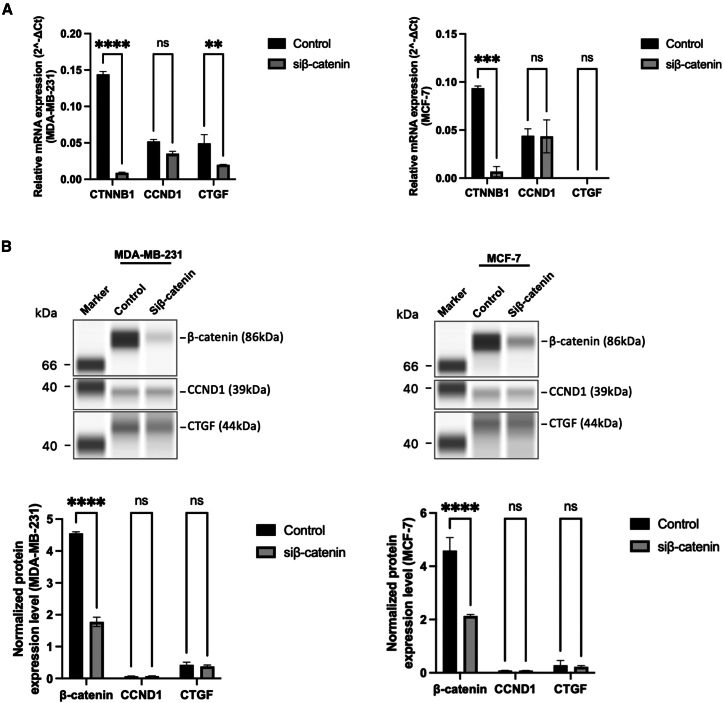
β-catenin knockdown reduces CTGF mRNA but not protein expression, and does not affect CCND1 expression (A) qPCR analysis showing efficient *CTNNB1* knockdown and the *CCND1* and *CTGF* transcript levels in MDA-MB-231 and MCF-7 cells. Data represent mean ± SD. *n* = 3 wells per condition. Statistical significance was assessed using two-way ANOVA followed by the Tukey post hoc test. ns: not significant, ∗∗*p* < 0.01, ∗∗∗*p* < 0.001, ∗∗∗∗*p* < 0.0001. (B) Western blots and quantification confirm reduced β-catenin protein following siRNA treatment, but no significant change in CCND1 or CTGF protein expression. These results suggest that β-catenin alone is insufficient to regulate YAP target genes in the absence of YAP. Data represent mean ± SD. *n* = 3 wells per condition. Statistical significance was assessed using two-way ANOVA followed by the Tukey post hoc test. ns: not significant, ∗∗∗∗*p* < 0.0001.

To determine whether β-catenin alone is sufficient to regulate canonical YAP target genes, we performed siRNA-mediated knockdown of *CTNNB1* in both MDA-MB-231 and MCF-7 cells. As shown in Figure 11A, qRT-PCR analysis confirmed effective knockdown of β-catenin transcripts in both cell lines, with a highly significant reduction in *CTNNB1* mRNA expression (∗∗*p* < 0.0001 in MDA-MB-231; ∗*p* < 0.001 in MCF-7). In MDA-MB-231 cells, the transcript level of *CTGF*, a downstream target of YAP, was also reduced following β-catenin depletion. In contrast, the transcript levels of *CCND1* remained unchanged in both cell lines (ns, not significant). These findings suggest that β-catenin contributes to CTGF expression, as its knockdown led to reduced CTGF transcript levels in MDA-MB-231 cells. However, CCND1 remained unaffected, indicating gene-specific regulation by β-catenin.

To validate these findings at the protein level, we assessed β-catenin, CCND1, and CTGF protein expression via capillary immunoblotting, as shown in Figure 11B. In both MDA-MB-231 and MCF-7 cells, β-catenin protein levels were drastically reduced upon siRNA treatment (∗∗*p* < 0.0001), confirming the efficiency of knockdown. While β-catenin knockdown reduced *CTGF* transcript levels in MDA-MB-231 cells, corresponding protein levels of CTGF remained unchanged (Figure 11B), suggesting post-transcriptional compensation. *CCND1* expression was unaffected at both mRNA and protein levels, indicating selective transcriptional regulation by β-catenin.

## Discussion

Our findings reveal a finely tuned, stiffness-dependent crosstalk between the Hippo and Wnt signaling pathways in metastatic breast cancer cells—a relationship that underlies how these cells sense and adapt to mechanical cues in their microenvironment.

In a mechanically stiff microenvironment, we observed a concerted activation of YAP and β-catenin: on rigid 2D substrates (∼32 kPa), MDA-MB-231 cells showed pronounced nuclear localization of both transcriptional regulators, whereas soft substrates (∼2 kPa) largely sequestered them in the cytoplasm (Figure 3). These results reinforce the concept that mechanical cues from the ECM can drive the nuclear translocation of YAP – a well-known mechanotransducer^32^ and extend this paradigm to β-catenin. Indeed, matrix rigidity is emerging as a potent activator of Wnt/β-catenin signaling even in the absence of Wnt ligands.^33^ A stiff ECM can signal through integrin-FAK and RhoGTPase pathways to stabilize β-catenin, which then translocates to the nucleus and even feeds back by upregulating WNT ligand expression.^33^ Consistent with this, we found that MDA-MB-231 cells on 32 kPa gels accumulated β-catenin in the nucleus alongside YAP. Such co-enrichment of YAP/TAZ and β-catenin has been noted as a general feature of mechanotransduction in tumors.^34^ It underscores that the YAP-β-catenin axis is a central node through which elevated ECM stiffness, a hallmark of aggressive tumors, can orchestrate oncogenic transcriptional programs.

A key discovery of this study is the mechanical context-dependence of YAP and β-catenin crosstalk. On compliant (soft) substrates, YAP appears to restrain Wnt/β-catenin activity, such that knocking down YAP increased β-catenin’s nuclear localization (Figure 4). In YAP-depleted cells on a 2 kPa substrate, β-catenin assumed a compensatory role: it remained or became nuclear and maintained the pro-migratory behavior, unless β-catenin was also silenced (Figure 6). This suggests that under low-tension conditions, metastatic cells can adapt by leveraging β-catenin signaling to uphold proliferative and migratory outputs when YAP is compromised. One mechanistic explanation is that YAP, when present in a soft context, might normally partake in sequestering β-catenin in the cytoplasm or otherwise limiting Wnt signaling. YAP and TAZ have been reported to bind β-catenin in the destruction complex, retaining β-catenin outside the nucleus and dampening Wnt pathway activation.^23^ Thus, on a compliant substrate where cytoskeletal tension and YAP activity are intrinsically low, loss of YAP could relieve this restraint and permit β-catenin to accumulate in the nucleus.

YAP silencing had a stronger effect on β-catenin nuclear localization and cell behavior under soft than stiff ECM conditions. This can be explained by mechanical saturation on rigid substrates: integrin-FAK-RhoA signaling already maximizes nuclear YAP and β-catenin, leaving little dynamic range for further change. In contrast, under soft conditions where mechanotransductive signals are sub-maximal, YAP restrains β-catenin within the destruction complex, and its knockdown “releases” β-catenin to the nucleus, partially compensating for YAP loss. Thus, YAP’s regulatory influence is most evident in compliant environments, whereas stiff matrices buffer against its depletion through parallel mechanotransduction pathways such as FAK-RhoA-SRF.^35^

Notably, we identified AXIN2 as a mechanosensitive Wnt target gene that exemplifies this interplay. AXIN2, a negative regulator of Wnt signaling, is itself a β-catenin/TCF target whose expression was sustained on stiff substrates even without YAP, presumably via β-catenin’s compensatory nuclear activity. In contrast, on soft substrates, AXIN2 expression dropped markedly with YAP knockdown (Figure 10), indicating that YAP is required to fully induce this Wnt feedback regulator under low mechanical tension. We propose that YAP acts as a crucial co-factor for Wnt pathway transcription in a soft environment, positioning AXIN2 as a downstream output of a YAP-dependent mechanotransduction cascade. This interpretation is in line with evidence that silencing YAP attenuates the transcription of Wnt/β-catenin target genes.^36^ YAP can modulate β-catenin-TCF-driven gene expression by multiple means, including direct binding to β-catenin or Wnt-regulatory elements.^28^ Therefore, the preservation of AXIN2 and CCND1 expression on stiff substrate (Figure 10) despite YAP loss underscores a context in which β-catenin alone can uphold certain gene programs, whereas the collapse of AXIN2 on stiff substrate without YAP highlights the mechanosensitive dependence of Wnt targets on YAP function.

Matrix stiffness influences Wnt/β-catenin signaling by altering β-catenin phosphorylation. On soft ECM, active GSK3β promotes phosphorylation at S33/S37/T41, marking β-catenin for degradation and limiting its nuclear availability.^24^ In contrast, stiff ECM activates integrin-FAK and Akt pathways that inhibit GSK3β, preventing this “destructive” phosphorylation and allowing β-catenin stabilization. Concurrently, PKA-mediated phosphorylation at S675, which enhances β-catenin nuclear translocation, may increase with stiffness through cAMP-PKA signaling. Together, these changes favor β-catenin accumulation and transcriptional activity on rigid matrices, providing a biochemical explanation for its enhanced nuclear localization under stiff conditions.^24^

Our study also underscores the importance of cytoskeletal tension and cell density in regulating YAP-β-catenin signaling. Pharmacological inhibition of non-muscle myosin II abrogated the nuclear translocation of both YAP and β-catenin (Figure 7), confirming that active actomyosin contractility is a prerequisite for their mechanosensitive activation. This result aligns with the canonical model of mechanotransduction wherein actin stress fibers and focal adhesions transmit stiffness cues to the nucleus, and loss of such tension (e.g., upon myosin II inhibition) leads to YAP inactivation and β-catenin degradation^33,39^.

Conversely, cell crowding (high cell confluency) attenuated β-catenin’s nuclear localization in response to YAP knockdown. At high cell density, cells engage contact inhibition signals: YAP is phosphorylated and cytoplasmic due to Hippo pathway activation, and β-catenin is tethered at adherens junctions by E-cadherin^40^. Under these conditions, the compensatory β-catenin nuclear response observed on soft substrates at low density was markedly suppressed (Figure 5). This finding highlights how cell-cell adhesion cues can override ECM-derived mechanical inputs, diminishing β-catenin’s transcriptional activity.

The prior studies have shown that YAP and β-catenin can form co-activator complexes at shared enhancers, with YAP enhancing β-catenin/TCF-mediated transcription and vice versa^41^. Our findings extend this model by demonstrating that in breast cancer cells under mechanical stress, this cooperation is highly context-dependent. To test the robustness of these findings in a more physiological setting, we incorporated a 3D spheroid model using Matrigel at 2, 4, and 8 mg/mL concentrations. In these 3D cultures, YAP knockdown consistently reduced proliferation and nuclear YAP levels (Figure 9). However, unlike in 2D culture, β-catenin nuclear localization decreased following YAP knockdown only at the lower Matrigel concentrations (2 and 4 mg/mL), and not at 8 mg/mL (Figure 9). This discrepancy suggests that in ultra-compliant environments, the compensatory β-catenin response observed in 2D soft matrices is suppressed, likely due to the absence of cytoskeletal tension required for nuclear translocation.

In conclusion, our study uncovers how metastatic breast cancer cells decode and respond to biomechanical signals through the YAP-β-catenin axis. Consistent with prior studies, our findings suggest that YAP-TEAD and β-catenin-TCF/LEF transcriptional programs are mechanically context-dependent. Under soft ECM conditions, YAP supports β-catenin/TCF-driven transcription, indicating TEAD-TCF synergy, whereas on stiff substrates, β-catenin-TCF activity becomes largely YAP-independent, with both pathways functioning in parallel. While our data establish a stiffness-gated YAP-β-catenin regulatory switch, the direct molecular basis remains to be confirmed. Future studies using chromatin immunoprecipitation (ChIP) and transcriptomic analyses will be essential to determine whether YAP and β-catenin co-occupy the AXIN2 promoter and co-regulate gene networks in a stiffness-dependent manner. Such genome-wide mapping will clarify how mechanical cues shape their cooperative transcriptional program and reveal potential therapeutic vulnerabilities within this mechanotransduction circuit.

### Limitations of the study

The goal of this study was to elucidate how extracellular matrix (ECM) stiffness regulates the interplay between YAP and β-catenin in metastatic breast cancer cells, and to provide experimental evidence supporting a stiffness-gated transcriptional relationship between these mechanosensitive pathways. While our findings offer insight into how mechanical cues shape YAP-β-catenin activity, several limitations must be considered when interpreting the results.

First, although we demonstrate stiffness-dependent changes in YAP and β-catenin localization and target gene regulation, the molecular mechanisms underlying this crosstalk remain incompletely resolved. Existing literature on how YAP functionally intersects with β-catenin under mechanical cues is fragmented and often limited to specific cellular contexts. Thus, while we synthesize key observations from different mechanobiology and signaling domains, the field lacks definitive, high resolution evidence on whether YAP and β-catenin physically interact, co-occupy chromatin regions, or regulate shared enhancers in a stiffness-dependent manner. Consequently, our conclusions regarding their cooperative transcriptional control, particularly for AXIN2, remain provisional.

Second, although we used two-dimensional substrates with controlled stiffness and three-dimensional Matrigel systems to model mechanical microenvironments, these platforms represent simplified reductions of the *in vivo* tumor niche. Breast tumors experience spatially heterogeneous, anisotropic, and dynamically remodeled ECM structures, influenced by stromal and immune cells. Our experimental systems do not fully capture these complexities. Additionally, Matrigel alters not only matrix stiffness but also biochemical composition, making it difficult to isolate the mechanical contributions from effects mediated by growth factors.

Third, most experimental manipulations relied on siRNA mediated knockdown of YAP and β-catenin. While effective, these approaches do not allow precise temporal control or full removal of target proteins. Off target effects cannot be fully excluded, and residual protein may preserve partial function. Future work employing CRISPR based disruption or inducible degron systems could provide more rigorous dissection of pathway dependencies.

Fourth, our assessment of Wnt pathway activity relied primarily on β-catenin nuclear localization and limited analysis of canonical target genes. As our results show, nuclear β-catenin does not uniformly translate into transcriptional activation, indicating that additional cofactors, including YAP itself, may be required under certain mechanical conditions. To fully understand stiffness-dependent transcriptional rewiring, genome wide assays such as RNA seq, ATAC seq, or ChIP seq will be necessary.

Fifth, although our work incorporated both luminal (MCF-7) and triple negative (MDA-MB-231) breast cancer cell lines, these two models do not encompass the broad heterogeneity of breast cancer. Subtype specific variations in cytoskeletal architecture, metabolic state, and intrinsic pathway mutations may alter mechanotransduction responses. Validation across additional models, including organoids derived from patients or *in vivo* systems, will be essential to determine the generalizability of our findings.

Finally, our three-dimensional spheroid experiments improve physiological relevance but remain limited in their ability to mimic real tumor environments. The spheroids lack directional matrix tension, interstitial flow, stromal cell interactions, and long term ECM remodeling, each of which is known to influence YAP and Wnt pathway activity. As such, the extent to which stiffness-dependent YAP-β-catenin coordination contributes to metastatic progression *in vivo* remains to be established.

Taken together, our study provides evidence for a mechanically gated YAP-β-catenin axis in metastatic breast cancer, but future work must incorporate more comprehensive mechanistic approaches and physiologically relevant models to establish the full scope and biological implications of this regulatory circuit.

## Resource availability

### Lead contact

Further information and requests for resources and reagents should be directed to and will be fulfilled by the Lead Contact, Dr. Fei Geng (gengf@mcmaster.ca).

### Materials availability

This study did not generate new unique reagents. All cell lines, siRNAs, and reagents are commercially available as listed above.

### Data and code availability

• Data: All data reported in this article are available within the main text and supplemental information. Raw images, unprocessed Western blots, and quantitative spreadsheets used for analysis are available from the Lead Contact upon request.
• Code: This article does not report original code. Any analysis scripts (ImageJ macros, Prism files) used for quantification are available from the lead contact upon request.
• Other items: Additional information required to reanalyze the data, including raw microscopy files, capillary immunoblot output files, and experimental protocols, is available from the lead contact upon request.

## Acknowledgments

This work was supported by grants from 10.13039/501100004489Mitacs, Grant/Award Number: IT35911; 10.13039/100008762Genome Canada Grant, Grant/Award Number: 2023-10. The authors express their gratitude to Dr. Juliet Daniel, who generously provided MDA-MB-231 cells for this research.

## Author contributions

F.G. and J.H. conceived and supervised the project. Z.H. and Y.W. performed cell culture of MCF-7 and MDA-MB-231 cells. Y.W. performed YAP knockdown, immunostaining, and image analysis. C.G. performed qRT-PCR and analyzed genetic data. Y.W. performed capillary electrophoresis, Western blotting, and data analysis. Y.W., Z.H., and F.G. wrote the article. All authors contributed to the article revision, read, and approved the submitted version.

## Declaration of interests

The authors declare no conflicts of interest.

## STAR★Methods

### Key resources table

**Table:** 

REAGENT or RESOURCE	SOURCE	IDENTIFIER
**Antibodies**		

Anti-YAP antibody	Cell Signaling Technology	Cat# 14074; RRID: AB_2650491
Anti-phospho-YAP (Ser127) antibody	Cell Signaling Technology	Cat# 4911; RRID: AB_10694635
Anti-β-catenin antibody	Cell Signaling Technology	Cat# 8480; RRID: AB_11127276
Anti-active β-catenin antibody (non-phospho Ser37/Thr41)	Cell Signaling Technology	Cat# 4270; RRID: AB_2797931
Alexa Fluor 488 Phalloidin	Invitrogen	Cat# A12379
Alexa Fluor 594 DNase I	Invitrogen	Cat# D12372
Alexa Fluor 568 secondary antibody	Thermo Fisher Scientific	Cat# A10042
DAPI	Thermo Fisher Scientific	Cat# D1306

**Chemicals, Peptides, and Recombinant Proteins**		

Collagen I (rat tail)	Thermo Fisher Scientific	Cat# A10483-01
CytoSoft Rigidity Plates (2 kPa, 32 kPa)	Advanced BioMatrix	Cat# 5190-02, 5190-32
Matrigel (standard & high concentration)	Corning	Cat# 354234, 354262
Blebbistatin	Sigma-Aldrich	Cat# B0560
Latrunculin A	Cayman Chemical	Cat# 10010630
alamarBlue Cell Viability Reagent	Invitrogen	Cat# DAL1025

**Critical Commercial Assays**		

DharmaFECT Transfection Reagent	Dharmacon	Cat# T-2001-01

**Experimental Models: Cell Lines**		

MCF-7 human breast cancer cell line	ATCC	HTB-22; RRID: CVCL_0031
MDA-MB-231 human breast cancer cell line	ATCC	HTB-26; RRID: CVCL_0062

**Oligonucleotides**		

siGENOME SMARTpool siYAP	Dharmacon	Cat# L-012200-00
siGENOME SMARTpool siβ-catenin	Dharmacon	Cat# L-003482-00
siGENOME Non-targeting Control siRNA	Dharmacon	Cat# D-001206-14

**Software and Algorithms**		

GraphPad Prism	GraphPad Software	Version 10; RRID: SCR_002798
ImageJ (FIJI)	NIH	RRID: SCR_002285
Gen5 Imaging Software	BioTek Instruments	https://www.biotek.com

### Experimental model and subject details

#### Cell lines

Human breast cancer cell lines MCF-7 (luminal A) and MDA-MB-231 (triple-negative) were used. Cells were cultured in DMEM supplemented with 10% FBS and 1% penicillin–streptomycin at 37°C and 5% CO_2_. Both lines were authenticated by STR profiling and confirmed mycoplasma-free before experiments.

### Method details

#### Substrate stiffness and cell culture

Cells were seeded onto collagen I-coated CytoSoft Rigidity Plates (2 kPa soft and 32 kPa stiff; Advanced BioMatrix).

Plates were pre-coated overnight at 4°C with 50 μg/mL collagen I in PBS. Cells were cultured 48 h before analysis.

#### siRNA transfection

At ∼50% confluence, cells were transfected with 25 nM SMARTpool siRNA (targeting YAP or β-catenin, or non-targeting control) using DharmaFECT reagent.

Knockdown efficiency was verified by capillary immunoblotting and qRT-PCR after 48 h.

#### 3D spheroid culture

MDA-MB-231 spheroids (10 000 cells/well) were generated in poly-HEMA-coated U-bottom 96-well plates.

After 24 h aggregation, spheroids were encapsulated in Matrigel (2, 4, or 8 mg/mL) to simulate stiffness gradients.

Following siRNA treatment, spheroids were cultured 48–96 h for bright-field imaging, alamarBlue assay, and immunofluorescence staining of YAP and β-catenin.

#### Immunofluorescence staining

Cells were fixed in 4% paraformaldehyde (15 min), permeabilized with 0.3% Triton X-100, and blocked in 4% BSA for 1 h.

Primary antibodies were incubated overnight at 4°C; fluorescent secondaries for 1 h at room temperature.

Nuclei were counterstained with DAPI and imaged using a Cytation 5 system (20× objective).

#### F-/G-actin quantification

Cells were stained with Phalloidin–Alexa Fluor 488 (F-actin, 1:3200) and DNase I–Alexa Fluor 594 (G-actin, 1:30).

Five random fields (*n* > 100 cells/condition) were analyzed with BioTek Gen5 software to calculate the F/G-actin ratio.

#### Nuclear/cytoplasmic intensity measurement

ImageJ (Fiji) was used to quantify fluorescence ratios. DAPI masks defined nuclear regions; cytoplasm was outlined manually.

The nuclear/cytoplasmic (N/C) ratio = mean nuclear intensity ÷ mean cytoplasmic intensity. ≥100 cells per condition from three independent replicates were analyzed.

#### Proliferation assay

Cells or spheroids were incubated with 10% alamarBlue for 2 h (2D) or 16 h (3D). Fluorescence (Ex 560 nm/Em 590 nm) was measured using a Tecan Infinite M200 Pro microplate reader.

#### Gap closure (migration) assay

Confluent monolayers were scratched using a sterile tip. Images were acquired at 0 h and 20 h using Cytation 5.

Migration rate = (Initial wound area − Final wound area)/20 h.

#### Protein quantification and capillary immunoblotting

Cells were lysed in 1% Triton X-100 buffer with protease inhibitors.

Protein concentration was determined by BCA assay, and capillary immunoblotting was performed using ProteinSimple Simple Western (Abby).

Antibody dilutions: YAP 1:50, p-YAP 1:50, β-catenin 1:250, active β-catenin 1:20; signals normalized to GAPDH or total protein.

#### qRT-PCR

Total RNA was isolated using RNeasy Mini Kit (Qiagen).

Reverse transcription and amplification used iTaq Universal SYBR Green One-Step Kit (Bio-Rad). Relative expression was calculated via ΔΔCt method using GAPDH as reference.

### Quantification and statistical analysis

All statistical analyses were performed using GraphPad Prism version 10. Unless otherwise specified, data are presented as mean ± SD, where the mean represents the center, and SD represents the dispersion of the data. The definition of n for each experiment is described in the corresponding figure legends.

Unless otherwise noted, comparisons between two groups were performed using a two-tailed unpaired Student’s *t* test, and comparisons among more than two groups were performed using a one-way or two-way ANOVA followed by Tukey post hoc test. A value of *p* < 0.05 was considered statistically significant.

For all Simple Western experiments, raw data and quantification were obtained using Compass for Simple Western software version 6.1 (BioTechne, Minneapolis, Minnesota, USA). Peak area values were used to quantify protein signal intensity. Protein intensities were normalized either to the total detected protein within each capillary or to GAPDH, as indicated in the figure legends (ProteinSimple, San Jose, California, USA).

For fluorescence-based single-cell quantification experiments, such as F/G-actin ratio and YAP or beta-catenin localization, images were acquired, and fluorescence intensities were measured using BioTek Gen5 software.

### Data availability statement

The original contribution data presented in the study are included in the article/Supporting Information; further inquiries can be directed to contact with the corresponding author.

## References

[1] Chiang A.C., Massagué J. (2008). Molecular basis of metastasis. N. Engl. J. Med. Overseas. Ed..

[2] Park M., Kim D., Ko S., Kim A., Mo K., Yoon H. (2022). Breast cancer metastasis: Mechanisms and therapeutic implications. Int. J. Mol. Sci..

[3] Riggio A.I., Varley K.E., Welm A.L. (2021). The lingering mysteries of metastatic recurrence in breast cancer. Br. J. Cancer.

[4] Roarty K., Echeverria G.V. (2021). Laboratory models for investigating breast cancer therapy resistance and metastasis. Front. Oncol..

[5] Valastyan S., Weinberg R.A. (2011). Tumor metastasis: Molecular insights and evolving paradigms. Cell.

[6] Jin X., Mu P. (2015). Targeting breast cancer metastasis. Breast Cancer.

[7] Yamaguchi H., Taouk G.M. (2020). A Potential Role of YAP/TAZ in the Interplay Between Metastasis and Metabolic Alterations. Front. Oncol..

[8] Nguyen L.T.S., Jacob M.A.C., Parajón E., Robinson D.N. (2022). Cancer as a biophysical disease: Targeting the mechanical-adaptability program. Biophys. J..

[9] Werneburg N., Gores G.J., Smoot R.L. (2020). The Hippo pathway and YAP signaling: Emerging concepts in regulation, signaling, and experimental targeting strategies with implications for hepatobiliary malignancies. Gene Expr..

[10] Deng B., Zhao Z., Kong W., Han C., Shen X., Zhou C. (2022). Biological role of matrix stiffness in tumor growth and treatment. J. Transl. Med..

[11] Ge H., Tian M., Pei Q., Tan F., Pei H. (2021). Extracellular matrix stiffness: New areas affecting cell metabolism. Front. Oncol..

[12] Venning F.A., Wullkopf L., Erler J.T. (2015). Targeting ECM Disrupts Cancer Progression. Front. Oncol..

[13] Wullkopf L., West A.K.V., Leijnse N., Cox T.R., Madsen C.D., Oddershede L.B., Erler J.T. (2018). & others. Cancer cells’ ability to mechanically adjust to extracellular matrix stiffness correlates with their invasive potential. Mol. Biol. Cell.

[14] Cai X., Wang K.C., Meng Z. (2021). Mechanoregulation of YAP and TAZ in cellular homeostasis and disease progression. Front. Cell Dev. Biol..

[15] Dupont S., Morsut L., Aragona M., Enzo E., Giulitti S., Cordenonsi M., Zanconato F., Le Digabel J., Forcato M., Bicciato S. (2011). others. Role of YAP/TAZ in mechanotransduction. Nature.

[16] Wada K.I., Itoga K., Okano T., Yonemura S., Sasaki H. (2011). Hippo pathway regulation by cell morphology and stress fibers. Development.

[17] Fu M., Hu Y., Lan T., Guan K.L., Luo T., Luo M. (2022). The Hippo signalling pathway and its implications in human health and diseases. Signal Transduct. Target. Ther..

[18] Hong W., Guan K.L. (2012). The YAP and TAZ transcription co-activators: Key downstream effectors of the mammalian Hippo pathway. Semin. Cell Dev. Biol..

[19] Reggiani F., Gobbi G., Ciarrocchi A., Sancisi V. (2021). YAP and TAZ are not identical twins. Trends Biochem. Sci..

[20] Cox T.R., Erler J.T. (2011). Remodeling and homeostasis of the extracellular matrix: implications for fibrotic diseases and cancer. Dis. Model. Mech..

[21] Chen W., Park S., Patel C., Bai Y., Henary K., Raha A., Mohammadi S., You L., Geng F. (2021). The migration of metastatic breast cancer cells is regulated by matrix stiffness via YAP signalling. Heliyon.

[22] Lin C.Y., Song X., Ke Y., Raha A., Wu Y., Wasi M., Wang L., Geng F., You L. (2022). Yoda1 enhanced low-magnitude high-frequency vibration on osteocytes in regulation of MDA-MB-231 breast cancer cell migration. Cancers (Basel).

[23] Astudillo P. (2020). Extracellular matrix stiffness and Wnt/β-catenin signaling in physiology and disease. Biochem. Soc. Trans..

[24] Liu J., Xiao Q., Xiao J., Niu C., Li Y., Zhang X., Zhou Z., Shu G., Yin G. (2022). Wnt/β-catenin signalling: function, biological mechanisms, and therapeutic opportunities. Signal Transduct. Target. Ther..

[25] Pai S.G., Carneiro B.A., Mota J.M. (2017). & others. Wnt/beta-catenin pathway: modulating anticancer immune response. J. Hematol. Oncol..

[26] Lin S.Y., Xia W., Wang J.C., Kwong K.Y., Spohn B., Wen Y., Pestell R.G., Hung M.C. (2000). & others. β-Catenin, a novel prognostic marker for breast cancer: Its roles in cyclin D1 expression and cancer progression. Proc. Natl. Acad. Sci. USA.

[27] Sefidbakht S., Saeedipour H., Saffar H., Mirzaian E. (2021). Determination of β-catenin expression in breast cancer and its relationship with clinicopathologic parameters. Asian Pac. J. Cancer Prev..

[28] Azzolin L., Panciera T., Soligo S., Enzo E., Bicciato S., Dupont S., Bresolin S., Frasson C., Basso G., Guzzardo V. (2014). others. YAP/TAZ incorporation in the β-catenin destruction complex orchestrates the wnt response. Cell.

[29] Konsavage W.M., Yochum G.S. (2013). Intersection of Hippo/YAP and Wnt/β-catenin signaling pathways. Acta Biochim. Biophys. Sin..

[30] Trejo-Solis C., Escamilla-Ramirez A., Jimenez-Farfan D., Castillo-Rodriguez R.A., Flores-Najera A., Cruz-Salgado A. (2021). others. Crosstalk of the Wnt/β-catenin signaling pathway in the induction of apoptosis on cancer cells. Pharmaceuticals.

[31] Chen W., Bai Y., Patel C., Geng F. (2019). Autophagy promotes triple negative breast cancer metastasis via YAP nuclear localization. Biochem. Biophys. Res. Commun..

[32] Lee J.Y., Chang J.K., Dominguez A.A., Lee H.P., Nam S., Chang J., Varma S., Qi L.S., West R.B., Chaudhuri O. (2019). YAP-independent mechanotransduction drives breast cancer progression. Nat. Commun..

[33] Du J., Zu Y., Li J., Du S., Xu Y., Zhang L., Jiang L., Wang Z., Chien S., Yang C. (2016). Extracellular matrix stiffness dictates Wnt expression through integrin pathway. Sci. Rep..

[34] Park Y., Lee D., Lee J.E., Park H.S., Jung S.S., Park D., Kang D.H., Lee S.I., Woo S.D., Chung C. (2024). The Matrix Stiffness Coordinates the Cell Proliferation and PD-L1 Expression via YAP in Lung Adenocarcinoma. Cancers (Basel).

[35] JIANG L., LI J., ZHANG C., SHANG Y., LIN J. (2020). YAP-mediated crosstalk between the Wnt and Hippo signaling pathways. Mol. Med. Rep..

[36] Andrews J.L., Kim A.C., Hens J.R. (2012). The role and function of cadherins in the mammary gland. Breast Cancer Res..

