# Supplementary material for: A stiffness-gated YAP-β-catenin axis orchestrates AXIN2 expression in metastatic breast cancer
Source: iScience. 2025 Dec 11;29(1):114405. doi: 10.1016/j.isci.2025.114405 (PMC12796759; doi:10.1016/j.isci.2025.114405)
Supplement: Document S1. Figures S1 and S2 [file mmc1.pdf]

iScience, Volume *29*

Supplemental information

A stiffness-gated YAP-β-catenin axis orchestrates AXIN2 expression in metastatic breast cancer

Yuning Wu, Zhi Su, Chang Ge, Shumaim Barooj, Jeremy A. Hirota, and Fei Geng

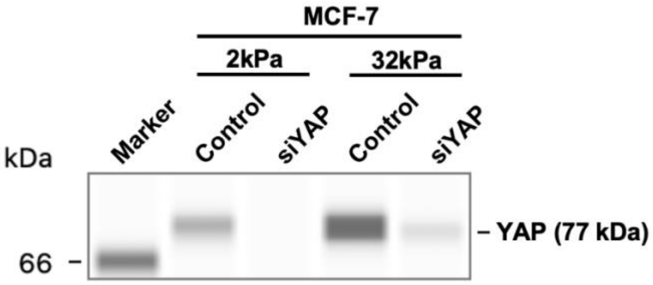

Figure S1: Validation of YAP knockdown efficiency in MCF-7 cells cultured on soft and stiff substrates.

Western blot analysis confirming YAP protein depletion following siYAP transfection in MCF-7 cells cultured on 2 kPa (soft) and 32 kPa (stiff) matrices. YAP (~77 kDa) levels were markedly reduced under both stiffness conditions compared to control siRNAtreated cells, verifying effective knockdown across mechanical contexts.

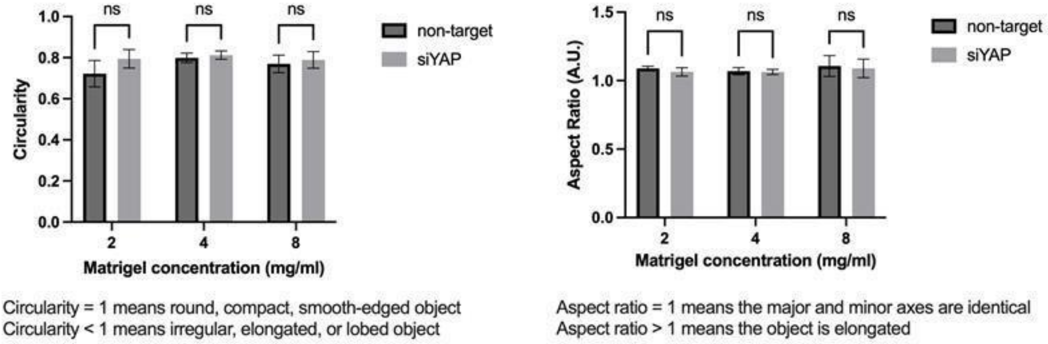

Figure S2: Quantitative analysis of spheroid morphology following YAP knockdown in 3D Matrigel cultures.

Circularity (left) and aspect ratio (right) of MDA-MB-231 spheroids cultured in 2, 4, or 8 mg/mL Matrigel were quantified using ImageJ Shape Descriptors (>100 spheroids per condition). No significant differences (*ns*, two-tailed *t*-test) were observed between non-target and siYAP-treated spheroids, indicating that YAP depletion does not alter spheroid compactness or symmetry across the tested stiffness range. Definitions for circularity and aspect ratio are shown below the plots. n = 3 wells per condition. Data are presented as mean ± SD; ns = not significant.
